# Comprehensive Behavioral Analysis of Cluster of Differentiation 47 Knockout Mice

**DOI:** 10.1371/journal.pone.0089584

**Published:** 2014-02-24

**Authors:** Hisatsugu Koshimizu, Keizo Takao, Takashi Matozaki, Hiroshi Ohnishi, Tsuyoshi Miyakawa

**Affiliations:** 1 Division of Systems Medical Science, Institute for Comprehensive Medical Science, Fujita Health University, Toyoake, Japan; 2 Core Research for Evolutional Science and Technology (CREST), Japan Science and Technology Agency, Kawaguchi, Japan; 3 Section of Behavior Patterns, Center for Genetic Analysis of Behavior, National Institute for Physiological Sciences, Okazaki, Japan; 4 Genetic Engineering and Functional Genomics Group, Frontier Technology Center, Kyoto University Graduate School of Medicine, Kyoto, Japan; 5 Laboratory of Biosignal Sciences, Institute for Molecular and Cellular Regulation, Gunma University, Maebashi, Japan; 6 Division of Molecular and Cellular Signaling, Department of Biochemistry and Molecular Biology, Kobe University Graduate School of Medicine, Kobe, Japan; 7 Department of Laboratory Sciences, Gunma University Graduate School of Health Sciences, Maebashi, Japan; Louisiana State University Health Sciences Center, United States of America

## Abstract

Cluster of differentiation 47 (CD47) is a member of the immunoglobulin superfamily which functions as a ligand for the extracellular region of signal regulatory protein α (SIRPα), a protein which is abundantly expressed in the brain. Previous studies, including ours, have demonstrated that both CD47 and SIRPα fulfill various functions in the central nervous system (CNS), such as the modulation of synaptic transmission and neuronal cell survival. We previously reported that CD47 is involved in the regulation of depression-like behavior of mice in the forced swim test through its modulation of tyrosine phosphorylation of SIRPα. However, other potential behavioral functions of CD47 remain largely unknown. In this study, in an effort to further investigate functional roles of CD47 in the CNS, CD47 knockout (KO) mice and their wild-type littermates were subjected to a battery of behavioral tests. CD47 KO mice displayed decreased prepulse inhibition, while the startle response did not differ between genotypes. The mutants exhibited slightly but significantly decreased sociability and social novelty preference in Crawley’s three-chamber social approach test, whereas in social interaction tests in which experimental and stimulus mice have direct contact with each other in a freely moving setting in a novel environment or home cage, there were no significant differences between the genotypes. While previous studies suggested that CD47 regulates fear memory in the inhibitory avoidance test in rodents, our CD47 KO mice exhibited normal fear and spatial memory in the fear conditioning and the Barnes maze tests, respectively. These findings suggest that CD47 is potentially involved in the regulation of sensorimotor gating and social behavior in mice.

## Background

Cluster of differentiation 47 (CD47), also referred to as integrin-associated protein (IAP), is a member of the immunoglobulin superfamily possessing a V-type immunoglobulin domain in its extracellular domain, five membrane-spanning domains and a short cytoplasmic tail [1]. CD47 functions as a ligand for the extracellular region of the transmembrane protein known as signal regulatory protein α (SIRPα; also known as SHPS-1, p84, and BIT) [2], [3]. SIRPα contains three Ig-like domains in its extracellular region and putative tyrosine phosphorylation sites in its cytoplasmic region [3], [4]. CD47 is expressed throughout the brain, with the regions in which it is abundant overlapping extensively with those enriched in SIRPα [5]–[7]. CD47 and SIRPα are both highly expressed in synapse-rich regions [2], [4], [6], and are considered to form a heterophilic complex to mediate bi-directional signaling between cells [2], [8], [9]. Binding of CD47 to SIRPα is required for the tyrosine phosphorylation of SIRPα [10], which is regulated by various receptor-type tyrosine kinases, including tropomyosin-related kinase B (TrkB), as well as the Src family kinases (SFKs) [4]. When SIRPα is phosphorylated, it then activates Src homology 2 domain–containing protein tyrosine phosphatase (Shp2) [11], [12], which is known to be involved in central nervous system (CNS) cell survival, differentiation, and cellular morphogenesis [13]–[15].

Our previous *in vitro* studies indicate that CD47 and SIRPα are involved in the regulation of brain-derived neurotrophic factor (BDNF)-dependent cell survival of CNS neurons, including cerebral cortical neurons, cerebellar granule neurons, and ventral mesencephalic dopaminergic neurons [16]–[19]. We also revealed that endogenous SIRPα is expressed at the surface of both axons and dendrites of cultured hippocampal neurons, and that endogenous CD47 is localized predominantly to the surface of dendrites [7]. Overexpression of CD47 and SIRPα in cultured mouse hippocampal neurons resulted in marked accumulation of those molecules at sites of contact between CD47-expressing dendrites and SIRPα-expressing axons [7]. In addition, expression of CD47 induced the phosphorylation of extracellular-signal-regulated kinase (ERK), which then led to dendritic outgrowth and up-regulation of glutamatergic synaptic transmission in rat cerebral cortical neurons [20]. These observations indicate that CD47 and SIRPα, which are differentially expressed on dendrites and axons, could generate a directional intercellular communication system that contributes to the formation and/or regulation of neural networks [7].

In a previous study, we also assessed the behavioral significance of CD47 and SIRPα, and revealed that these molecules are involved in the regulation of immobility in the Porsolt forced swim test in mice [21]. In particular, both mice expressing a form of SIRPα that lacks most of the cytoplasmic region (SIRPα mutant mice) and CD47 knockout (KO) mice exhibited prolonged immobility in the forced swim test, suggesting that depression-like behavior, as assessed by this test, is increased in the mice; however, in SIRPα mutant mice, no significant effects were detected in the tail suspension test [21]. It was determined that phosphorylation of the cytoplasmic region of SIRPα, which requires binding of CD47 to SIRPα, was induced by forced swim stress, but not tail suspension stress (Table S1) [21]. These findings indicate that the CD47-SIRPα signal regulates depression-like behavior in the response to stress that leads to phosphorylation of SIRPα [21]. It was also previously reported that CD47 mRNA levels are significantly higher in rats showing good retention performance in an inhibitory avoidance learning paradigm than in rats exhibiting poor retention performance [22], and that memory retention of CD47 KO mice is significantly impaired in an inhibitory avoidance learning paradigm [23] (Table S1). Those observations suggest that CD47 may be involved in the regulation of fear memory in rodents [22], [23]. However, other aspects of CD47′s effects on behavior remain largely unknown.

The objective of the present study was to further investigate the behavioral phenotypes of CD47 KO mice by subjecting the mutants and their wild-type littermates to a comprehensive behavioral test battery. The test battery included wire hang, grip strength, rotarod, hot plate, gait analysis, Crawley’s three-chamber social approach, social interaction (home cage/novel environment), open field, startle response/prepulse inhibition (PPI), light/dark transition, elevated plus maze, tail suspension, fear conditioning, and Barnes maze tests. A series of tests revealed novel phenotypes of CD47 KO mice including a decreased PPI and abnormal social interaction.

## Results

### 1. General Health, Motor Function, and Nociception in CD47 KO Mice

**Figure 1:**
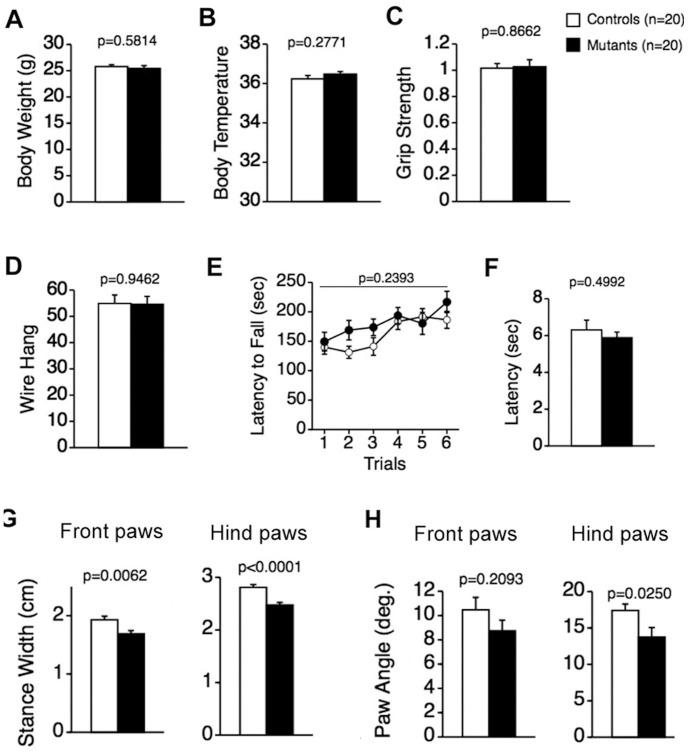
Physical characteristics, neuromuscular strength, motor coordination, nociception, and gait in CD47 KO and wild-type mice. No significant differences between genotypes were detected in body weight (A), body temperature (B), grip strength (C), or latency to fall in the wire hang test (D). (E) Likewise, no significant differences between the genotypes were observed in latency to fall from the rotarod (F) or latency to the first paw response in the hot plate test. CD47 KO mice, n = 20; wild-type mice, n = 20. (G–H) CD47 KO mice were also subjected to gait analysis. (G) The mutants showed significant decreases in stance width for front and hind paws compared to wild-type mice. (H) Paw angles for hind paws, but not for front paws, were significantly narrower in the mutants than in wild-type mice. CD47 KO mice, n = 19; wild-type mice, n = 20.

There were no significant differences between CD47 KO mice and their wild-type littermates in body weight (Figure 1A; F_1,38_ = 0.309, *p* = 0.5814) or body temperature (Figure 1B; F_1,38_ = 1.216, *p = *0.2771). Measures of neuromuscular strength and motor coordination learning likewise did not differ between the genotypes. Specifically, grip strength (Figure 1C; F_1,38_ = 0.029, *p* = 0.8662), latency to fall in the wire hang test (Figure 1D; F_1,38_ = 0.005, *p* = 0.9462), and latency to fall off the rotarod (Figure 1E; F_1,38_ = 1.429, *p* = 0.2393) did not differ significantly between CD47 KO and wild-type mice. There was also no significant difference in nociception between CD47 KO and wild-type mice as measured by the first paw response the hot plate test (Figure 1F; F_1,38_ = 0.466, *p* = 0.4992). In addition, brain weight did not significantly differ between genotypes (wild-type mice, 0.478 ± 0.005 g, n = 14; CD47 KO mice, 0.488 ± 0.004 g, n = 12; Student’s *t*-test, p = 0.1338).

The gait pattern of CD47 KO mice was also examined. CD47 KO mice showed significant reductions in stance width for both front and hind paws compared to wild-type mice (front paws, F_1,37_ = 8.410, *p = *0.0062, Figure 1G; hind paws, F_1,37_ = 21.075, *p* < 0.0001, Figure 1G). In addition, there was a significant difference in paw angles for hind paws (Figure 1H; F_1,37_ = 5.462, *p* = 0.0250) but not for front paws between the genotypes (Figure 1H; F_1,37_ = 1.633, *p* = 0.2093). Specifically, the stance widths of front and hind paws in CD47 KO mice were narrower than those of wild-type mice, possibly reflecting improved postural adjustments for stability, similar to those previously demonstrated during recovery from injury after a locomotor training paradigm [24]. Paw placement angles for the hind paws were also narrower in CD47 KO mice than in wild-type mice. It is known that more open angles of the hind paws are associated with ataxia, spinal cord injury, and demyelinating disease [25]. These findings suggest that CD47 is involved in the regulation of gait in mice and that deficiency of CD47 may in fact lead to improved motor coordination.

### 2. Decreased Prepulse Inhibition (PPI) in CD47 KO Mice

**Figure 2:**
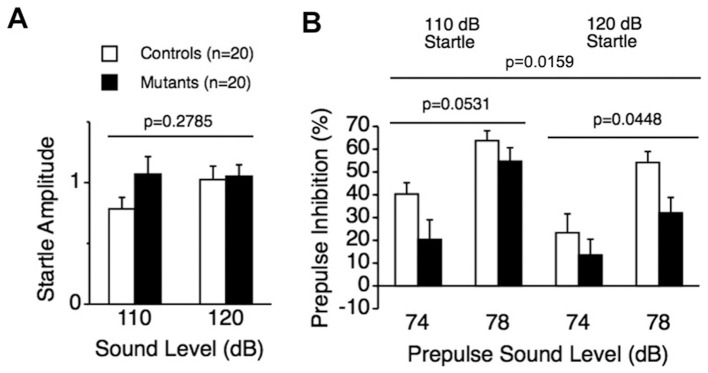
Normal startle response and decreased PPI in CD47 KO mice. (A) CD47 KO mice demonstrated normal acoustic startle responses to the 110 dB and 120 dB startle stimuli. (B) CD47 KO mice showed a significant reduction in PPI for the 74 and 78 dB prepulse sound level compared to wild-type mice. PPI in the mutants was significantly lower than that in wild-type mice when the startle stimulus was 120 dB, whereas only a trend for reduced PPI was observed in the mutants when startle stimulus was 110 dB. CD47 KO mice, n = 20; wild-type mice, n = 20.

To assess whether deficiency of CD47 affects sensorimotor gating in mice, CD47 KO mice were subjected to startle response/PPI tests. CD47 KO mice displayed normal acoustic startle responses to the 110 dB and 120 dB startle stimuli (Figure 2A; genotype effect, F_1,38_ = 1.209, *p* = 0.2785, two-way repeated measures ANOVA) but reductions in PPI compared to wild-type mice (Figure 2B; genotype effect, F_1,38_ = 18.868, *p* = 0.0159, two-way repeated measures ANOVA). PPI in the mutants was significantly lower than that of wild-type mice when the startle stimulus was 120 dB (Figure 2B; genotype effect, F_1,38_ = 4.304, *p* = 0.0448, two-way repeated measures ANOVA) and tended to be lower when the startle stimulus was 110 dB (Figure 2B; genotype effect, F_1,38_ = 3.984, *p* = 0.0531, two-way repeated measures ANOVA). These results suggest impaired sensorimotor gating in CD47 KO mice.

### 3. Abnormal Social Behaviors in CD47 KO Mice

**Figure 3:**
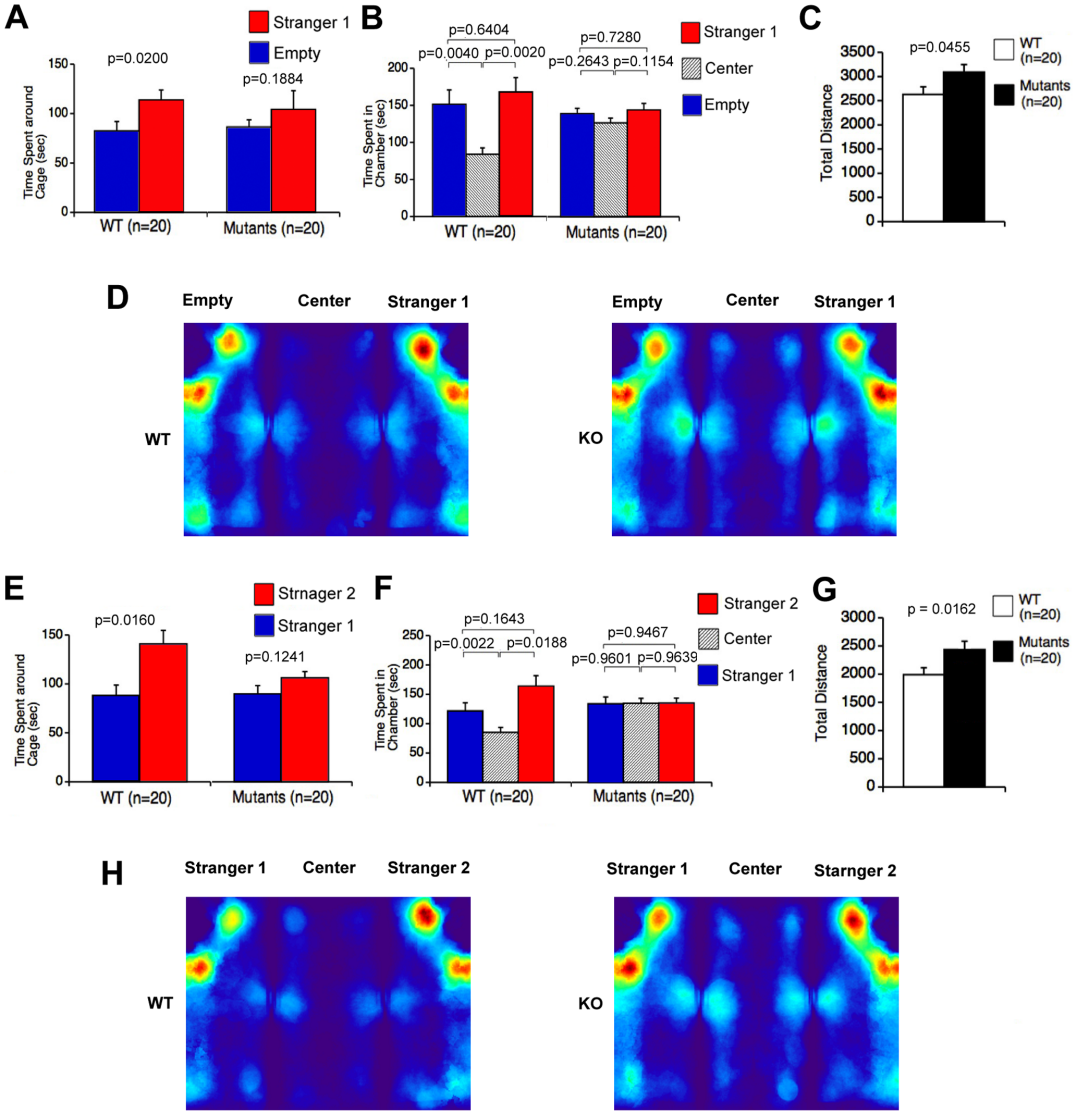
Abnormal social behavior of CD47 KO mice in Crawley’s sociability and social novelty preference test. The sociability test was performed first (A–D). (A) CD47 KO mice did not show significant differences between time spent around cage with stranger 1 and time spent around the empty cage. By comparison, wild-type mice spent more time around the cage with the stranger 1 than around the empty cage. (B) CD47 KO mice displayed no significant differences in time spent among the three chambers, whereas wild-type mice spent less time in the center chamber than in the other two chambers. (C) Distance traveled by CD47 KO mice was significantly longer than that of wild-type mice. (D) All images of each mouse were superimposed, and averaged images for the traces of wild-type mice (left, WT) and CD47 KO mice (right, KO) in the sociability test are presented. Following completion of the sociability test, the social novelty preference test was carried out (E–H). (E) The mutants exhibited no significant differences between time spent around the cage with stranger 1 (familiar side) and time spent around the cage with stranger 2 (stranger side). However, wild-type mice spent significantly less time in the familiar side (stranger 1) than in the stranger side (stranger 2). (F) No significant differences were detected in time spent in the three chambers in CD47 KO mice, while wild-type mice spent less time in the center chamber than in the other chambers. (G) Distance traveled by CD47 KO mice was significantly longer than distance traveled by wild-type mice. (H) All images of each mouse were superimposed and averaged images for the traces of wild-type mice (left) and CD47 KO mice (right) in the social novelty preference test are shown. CD47 KO mice, n = 20; wild-type mice, n = 20.

CD47 KO mice were also subjected to Crawley’s three-chamber social approach test. This test allows for both a sociability and a social novelty preference test. Importantly, the sociability assay assesses social behavior independently of differences in locomotor activity because the social preference of mice can be quantified by comparing the time spent around a wire cage containing a stranger mouse versus the time spent around an empty cage [26]. In the sociability test, there was no significant genotype effect on ratio of time spent around cage with stranger to total time spent with both cages (Mann-Whitney U-test, *p* = 0.4819). Next, we compared time spent around cage with stranger and that of empty cage within each genotype. There were no significant differences between time spent around the cage with stranger 1 and time spent around the empty cage in the mutants (Figure 3A; *t*_19_ = 1.364, *p* = 0.1884), while in wild-type mice, time spent around the cage with stranger 1 was significantly longer than time spent around the empty one (Figure 3A; *t*_19_ = 3.584, *p* = 0.0200). Moreover, CD47 KO mice showed no significant differences in time spent among the three chambers (Figure 3B, D; stranger 1 side versus empty cage side, *t*_19_ = 0.353 *p* = 0.7280; stranger 1 side versus center, *t*_19_ = 1.650, *p* = 0.1154; center side versus empty cage side, *t*_19_ = −1.150, *p* = 0.2643). On the other hand, wild-type mice spent less time in the center chamber than in the other two chambers (Figure 3B, D; stranger side versus empty side, *t*_19_ = 0.475, *p = *0.6404; center versus stranger side, *t*_19_ = 3.584, *p* = 0.0020; center versus empty side, *t*_19_ = −3.268, *p = *0.0040). Ratio of time spent in stranger side to center area for CD47 KO mice was significantly smaller than that for wild type mice (Mann-Whitney U-test, *p* = 0.0041). In the sociability test, CD47 KO mice also showed small but significantly greater values for total distance traveled compared to that of their wild-type littermate controls (Figure 3C; F_1,38_ = 4.319, *p* = 0.0445). Next, the social novelty preference test was performed. In the social novelty preference test, social behavior was evaluated by contact with the first, already-investigated mouse (stranger 1/familiar side) and a novel mouse (stranger 2/stranger side) in the wire cage. There were no significant differences in time spent around the cage with stranger 1 (familiar side) versus stranger 2 (stranger side) in the mutants (Figure 3E; *t*_19_ = 1.609, *p* = 0.1241). In contrast, wild-type mice spent significantly more time around the familiar side than the stranger side (Figure 3E; *t*_19_ = 2.643, *p* = 0.0160). Genotype comparison revealed that the mutants spent significantly less time around the cage with stranger 2 (stranger side) than did the wild-type mice (genotype effect, F_1,38_ = 5.192, *p* = 0.0284). No significant genotype effect was detected on ratio of time spent with stranger 2 / total time spent with both cages (Mann-Whitney U-test, *p* = 0.1762). In CD47 KO mice, no significant differences were detected in time spent among the three chambers (Figure 3F, H; familiar side versus stranger side, *t*_19_ = 0.068, *p* = 0.9467; center vs. familiar side, *t*_19_ = 0.046, *p* = 0.9639; center vs. stranger side, *t*_19_ = 0.051, *p* = 0.9601). In contrast, wild-type mice spent significantly less time in the center chamber than in the other chambers (Figure 3F, H; familiar side versus stranger side, *t*_19_ = 1.447, *p* = 0.1643; center versus familiar side, *t*_19_ = 3.546, *p* = 0.0022; center versus stranger side, *t*_19_ = −2.569, *p* = 0.0188). Ratio of time spent in stranger 2 side to center area for CD47 KO mice was smaller than that for wild type mice (Mann-Whitney U-test, *p* = 0.0041). The mutants also showed significantly greater values for total distance traveled than wild-type mice (Figure 3G; F_1,38_ = 6.329, *p* = 0.0162).

**Figure 4:**
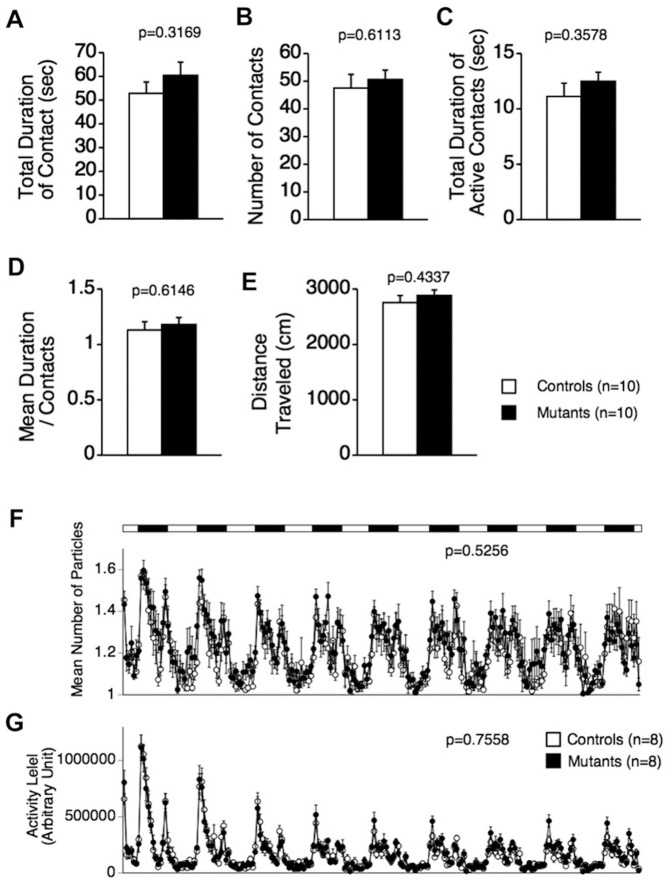
Normal social behaviors of CD47 KO mice in a novel environment and the home cage. (A–E) In the social interaction test performed in a novel environment, there were no significant differences between the genotypes in total duration of contacts (A), number of contacts (B), total duration of active contacts (C), mean duration per contact (D), or distance traveled (E). CD47 KO mice, n = 10 pairs; wild-type mice, n = 10 pairs. (F, G) Social interaction in the home cage was monitored over a 9-day period. There were no significant differences between the genotypes in the mean numbers of particles, i.e., whether 1 (mice together) or 2 (mice apart) subjects were detected (F) or activity level (G). CD47 KO mice, n = 8 pairs; wild-type mice, n = 8 pairs.

In an alternative version of the social interaction test in which two mice move freely and can have direct interactions in a novel environment for 10 min, no significant differences were detected between the genotypes in total duration of contacts (Figure 4A; F_1,18_ = 1.060, *p* = 0. 3169), number of contacts (Figure 4B; F_1,18_ = 0.268, *p* = 0. 6113), total duration of active contacts (Figure 4C; F_1,18_ = 0.891, *p* = 0. 3578), mean duration per contact (Figure 4D; F_1,18_ = 0.263, *p = *0. 6146), or distance traveled (Figure 4E; F_1,18_ = 0.641, *p* = 0. 4337). Social interaction was also monitored in the home cage over a 9-day period. There were no significant differences between the genotypes in the mean numbers of particles (Figure 4F; F_1,14_ = 0.424, *p* = 0.5256) or activity level (Figure 4G; F_1,14_ = 0.101, *p* = 0.7558). Thus, although no abnormalities in social behavior of the mutants were detected in the social interaction tests in which mice had direct contact under freely moving settings, significantly reduced social behavior was observed in Crawley’s three-chamber social approach test. These findings suggest that a CD47 deficiency may induce mild impairments in sociability and social novelty preference in mice.

### 4. Normal Anxiety-like and Depression-like Behaviors in CD47 KO Mice

**Figure 5:**
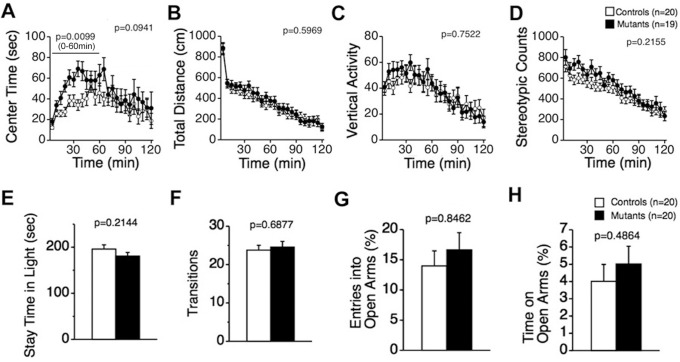
Normal locomotor activity in CD47 KO mice. In the open field test, (A) CD47 KO mice stayed significantly longer in the center of area of the apparatus than wild-type mice during the earlier part of the experimental period, but not over the entire experimental period. No significant differences were detected in total distance traveled (B), vertical activity (C), or stereotypic counts (D). CD47 KO mice, n = 19; wild-type mice, n = 20. CD47 KO mice and wild-type mice were also subjected to the light/dark transition test (E–F). Time spent in the light box (E) and number of transitions (F) did not significantly differ between genotypes. CD47 KO mice, n = 20; wild-type mice, n = 20. The elevated plus maze test was also performed (G–H). There were no significant differences between the genotypes in the number of entries to the open arms (G) or time spent on the open arms (H). CD47 KO mice, n = 20; wild-type mice, n = 20.

Anxiety-like behaviors of CD47 KO mice were evaluated in the open field, light/dark transition, and elevated plus-maze tests. In the open field test, CD47 KO mice tended to spend longer in the center area compared to wild-type mice over the entire experimental period (Figure 5A; 0–120 min, F_1,37_ = 2.952, *p* = 0.0941), and the mutants showed an increase in center time during the earlier part of the experimental period (Fig. 5A; 0–60 min, F_1,37_ = 7.390, *p* = 0.0099). Since increased center time was only seen in the earlier part of the experimental period, it is possible that a deficiency in CD47 may lead to decreased anxiety in a novel environment in these mice. On the other hand, there were no significant differences in total distance traveled (Figure 5B; F_1,37_ = 0.285, *p* = 0.5969), vertical activity (Figure 5C; F_1,37_ = 0.101, *p* = 0.7522), or stereotypic behavior (Figure 5D; F_1,37_ = 1.588, *p* = 0.2155). In the light/dark transition test, no significant differences were observed between the genotypes with respect to time spent in the light box (Figure 5E; F_1,38_ = 1.594, *p* = 0.2144) or number of transitions (Figure 5F; F_1,38_ = 0.164, *p* = 0.6877). In the elevated plus-maze test, there were no significant differences between genotypes in entries to the open arms (Figure 5G; F_1,38_ = 0.038, *p* = 0.8462), or time spent on the open arms (Figure 5H; F_1,38_ = 0.494, *p* = 0.4864). Thus, anxiety-like behaviors did not significantly differ between genotypes.

**Figure 6:**
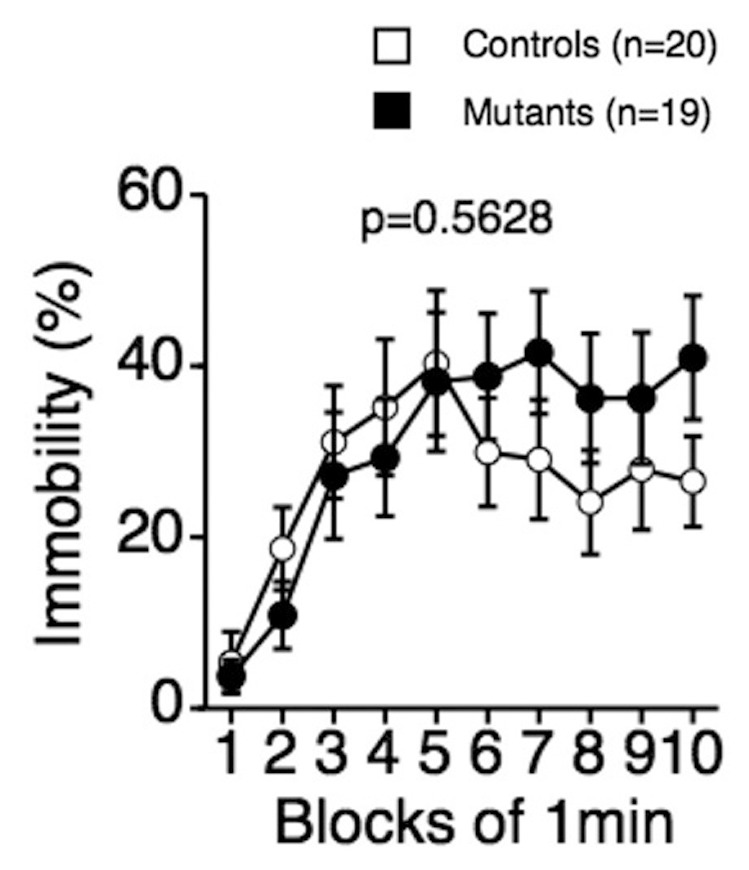
Normal depression-like behavior of CD47 KO mice in the tail suspension test. There was no significant genotype effect for the percentage of immobility time in the tail suspension test. CD47 KO mice, n = 19; wild-type mice, n = 20.

CD47 KO mice were also subjected to the tail suspension test to assess depression-like behavior. No significant effect of genotype was found for percent time immobile (Figure 6; F_1,37_ = 0.341, *p* = 0.5628). Thus, in the tail suspension test, in which phosphorylation of SIRPα is known not to be induced [21], no behavioral abnormalities were detected in CD47 KO mice.

### 5. Normal Fear and Spatial Memory in CD47 KO Mice

**Figure 7:**
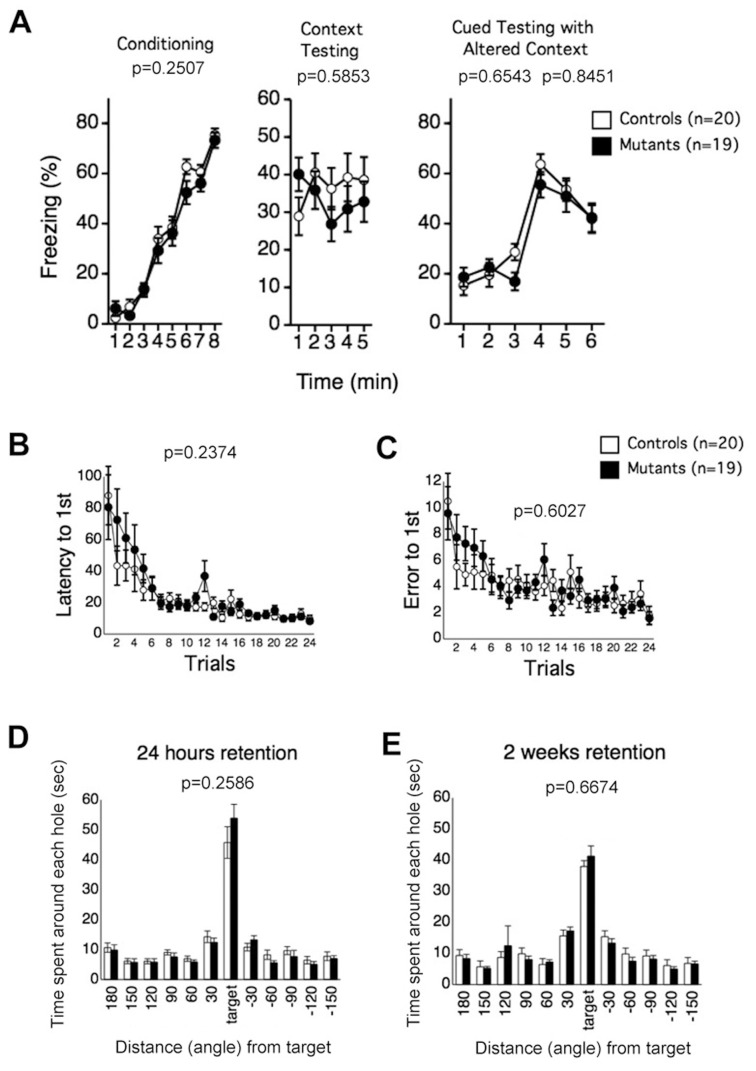
Normal fear and spatial memory in CD47 KO mice. (A) In the fear conditioning test, no significant differences between genotypes were observed in freezing during conditioning, context testing, or cued testing in an altered context. CD47 KO mice, n = 19; wild-type mice, n = 20. (B–E) In the training session of the Barnes maze test, no significant differences between the genotypes were detected in the latency to the 1^st^ hole (the escape box) (B), or error to the 1^st^ hole in the probe test (C). In a retention test performed either 24 h (D) or 2 weeks (E) after the last training session, there were no significant differences between the genotypes in time spent around the hole where the escape box had been placed (target). CD47 KO mice, n = 19; wild-type mice, n = 20.

Finally, learning and memory of CD47 KO mice was assessed in the fear conditioning and Barnes maze tests. In the fear conditioning test, no significant differences between the genotypes were detected in the amount of freezing after footshocks in the conditioning phase (Figure 7A; F_1,37_ = 1.361, *p = *0.2507). Contextual freezing of the mutants also did not significantly differ from that of wild-type mice 1 day after training (Figure 7A; F_1,37_ = 0.044, *p* = 0.5853). Freezing before or after the auditory cue as measured in an altered context was also not significantly different across the genotypes (Figure 7A; F_1,37_ = 0.204, *p* = 0.6543 [before tone]; F_1,37_ = 0.039, *p* = 0.8451 [after tone]). In the Barnes maze test, CD47 KO mice showed no significant differences in time required to reach the escape box (the 1^st^ hole) (Figure 7B; F_1,37_ = 1.443, *p* = 0.2374) or number of errors (Figure 7C; F_1,37_ = 0.276, *p* = 0.6027) during the training session. There were also no significant differences between the genotypes in time spent around the hole where the escape box had been placed in probe tests performed either 24 h (Figure 7D; F_1,37_ = 1.317, *p* = 0.2586) or 2 weeks (Figure 7E; F_1,37_ = 0.188, *p* = 0.6674) after the last training session. These findings indicate normal memory acquisition and retention in the mutants. The present study thus failed to detect impairments of CD47 KO mice in learning and memory.

## Discussion

In this study, we assessed the behavioral phenotypes of CD47 KO mice with our comprehensive behavioral test battery. We have summarized the behavioral phenotypes of CD47 KO mice, CD47 antisense-injected rats [22], and SIRPα mutant mice [21], as identified in the present and previous studies [23], in Table S1. While the present study revealed that the lack of CD47 did not lead to significant abnormalities in overall health, a few physical and behavioral characteristics were newly identified. First, in the startle response/PPI tests, while the acoustic startle response of CD47 KO mice did not significantly differ from that of their wild-type littermate mice, the mutants exhibited mild but significant reductions in PPI, suggesting that CD47 is involved in the regulation of sensorimotor gating in mice.

Second, in Crawley’s three-chamber social approach test, the mutants showed slight reductions in sociability and social novelty preference, indicating that CD47 is involved in the regulation of social behavior. On the other hand, in social interaction tests in which pairs of subject animals could interact directly in a novel environment or their home cage, no abnormalities were detected in either social behavior or locomotor activity of the mutants. It is possible that these discrepant findings in different social interaction assays are due to the assays’ differential requirements for intact olfactory function. It is reported that when animals have olfactory impairment, abnormal social behaviors can be observed in Crawley’s three-chamber social approach test, in which mice must spend time near animals that are isolated in wire cages and not directly accessible [27]. Therefore, the possibility cannot be excluded that the decreased sociability and social novelty preference in this test were caused by an olfactory impairment in the mutant mice. Further behavioral or physiological analyses would be needed to address whether CD47 KO mice have normal olfaction.

Third, in the gait analysis the mutants demonstrated narrower stance widths of the front and hind paws, and narrower paw placement angles for the hind paws. These findings indicate that CD47 is involved in motor coordination and that CD KO may lead to improved postural adjustments and stability [24], [25]. Additional histological and electrophysiological analyses in brain areas such as the cerebellum, basal ganglia, and motor cortex, would be important to assess the role of CD47 in motor coordination in mice. Additionally, in the open field test, CD47 KO mice stayed significantly longer in the center of the field during the earlier part of the experimental period, but not over the entire experimental period, indicating that the mutants show decreased anxiety-like behavior in a novel environment. These results point to the possibility that CD47 is involved in the regulation of anxiety in a novel environment.

In the tail suspension test, CD47 KO mice exhibited no impairments in depression-like behavior. In our previous study, we revealed that forced swimming stress, but not tail suspension stress, led to CD47-dependent tyrosine phosphorylation of SIRPα in the brain of mice [21]. In an earlier study, both CD47 KO mice and SIRPα mutant mice displayed increased depression-like behaviors in the forced swimming test, while there were no changes in depression-like behavior of SIRPα mutant mice in the tail suspension test (Table S1) [21]. Our previous findings indicate that CD47 and SIRPα are not involved in the regulation of depression-like behaviors when stress does not cause tyrosine phosphorylation of SIRPα in the brain [21], and this is consistent with the present observation that CD47 KO mice demonstrated normal performance in a test that does not lead to CD47-dependent tyrosine phosphorylation of SIRPα [21]. It would be of interest to address whether tyrosine phosphorylation of SIRPα is also involved in the regulation of other behaviors.

The present study also failed to detect impairments of learning and memory in CD47 KO mice in the fear conditioning and Barnes maze tests. On the other hand, in an inhibitory avoidance learning paradigm, previous studies demonstrated that CD47 deficiency led to an impairment of learning and memory in CD47 KO mice and CD47 antisense-injected rats in an inhibitory avoidance learning paradigm (Table S1) [22], [23]. There are several possible explanations for these inconsistent results. First, differences in species, genetic backgrounds, and age may cause differences in learning and memory performance. In the paper by Chang et al., the CD47-null allele was backcrossed from CD47^+/-^ 129sv/eg mice into Balb/cJ [23], whereas in the present study the mutants tested were CD47 KO mice that were backcrossed to C57BL/6J. Huang et al. used rats in their study [22]. In addition, the mutants in the paper by Chang et al. were subjected to the inhibitory avoidance test at 14–21 weeks of age [23], while the Barnes maze and fear conditioning tests were performed in the present study using mice at 39–48 and 43–52 weeks of age, respectively. There is therefore the possibility that CD47 KO leads to deficits in learning and memory performance only in young adult mice. Second, it is also possible that experiences during the behavioral test battery carried out prior to the Barnes maze and fear conditioning tests caused the different genotype effects on learning and memory performance. Third, there are known differences between the mechanisms of fear memory consolidation as it occurs in assays of fear conditioning and inhibitory avoidance learning [28], [29]. For instance, pharmacological inactivation of GABA_A_ receptors in the lateral and basal nuclei of the amygdala indicate that the amygdala could play an essential role in the acquisition of fear conditioning, and contributes to the modulation of memory consolidation of inhibitory avoidance, but not of fear conditioning [28]. It is therefore possible that CD47 plays a role in regulation of amygdala-dependent learning and memory via the GABA_A_ receptor-mediated signaling. Lastly, the passive avoidance test could yield results that are inconsistent with those in the fear conditioning test. For example, muscarinic acetylcholine receptor M2 KO mice in one study demonstrated no impairments in fear conditioning, despite the fact that M2 KO led to impairment in the acquisition of a passive avoidance task [30]. In another example, GSK-3α KO mice exhibited a deficit in fear conditioning, but their learning and memory performance in the passive avoidance test was normal [31]. Additional studies, including histological and electrophysiological analyses, will be required to elucidate whether and how CD47 contributes to the regulation of learning and memory in mice. Using Hematoxylin & Eosin (HE) staining, we failed to detect obvious differences in the overall structure of the hippocampus between the genotypes (data not shown). In addition to the hippocampus, further precise analyses would be needed in the amygdala and other brain areas.

It is notable that a few studies reported that expression of CD47 is dysregulated in patients with psychiatric disorders including schizophrenia and attention deficit hyperactivity disorder (ADHD) [32]–[34]. In particular, the expression of CD47 is down-regulated in Brodmann area 46 in the prefrontal cortex [32], and up-regulated in whole blood of patients with schizophrenia [33]. In patients with ADHD, a rare copy number variation (CNV) was identified in the proximal 5′-upstream region of the CD47 gene [34]. The present study suggests that CD47 deficiency in mice leads to decreased PPI, and, potentially, abnormal social behavior, both of which are considered to be behavioral abnormalities relevant to schizophrenia [35]. Abnormal social behavior is also thought to be a trait related to ADHD [36]. On the other hand, this study failed to detect the impaired working memory in CD47 KO mice that is often observed in the patients with schizophrenia [35] and ADHD [36]. CD47 KO mice may thus recapitulate selected aspects of schizophrenia and ADHD, suggesting that CD47 may represent a new therapeutic target for these conditions.

In conclusion, the present study suggests that CD47 is potentially involved in the regulation of sensorimotor gating, social behavior, and gait in mice. It remains unclear how the deficiency of CD47 leads to the behavioral phenotypes of the mutants. Further studies are required to determine the sites of the brain, developmental stages, and possible mechanisms that contribute importantly to produce the behavioral phenotypes of CD47 KO mice.

## Methods

### Ethical Statement

All animal care, behavioral testing procedure, and animal experiments were approved by the Animal Research Committee, Graduate School of Medicine, Kyoto University (Permit No., MedKyo 09539) and the Institutional Animal Care and Use Committee of Fujita Health University (Permit No., I0741), based on the Law for the Humane Treatment and Management of Animals (2005) and the Standards Relating to the Care and Management of Laboratory Animals and Relief of Pain (2006). Every effort was made to minimize the number of animals used.

### Animals and Experimental Design

CD47 KO mice were generated as previously reported [37]. They were backcrossed onto a C57BL/6J line for at least ten generations. Mice were group housed (2–4 mice per cage) in a room with a 12-h light/dark cycle (lights on at 7∶00 a.m.) with access to food and water ad libitum. Behavioral testing was performed between 9∶00 a.m. and 6∶00 p.m. except where otherwise indicated. Before testing each animal, each apparatus was cleaned with diluted sodium hypochlorite solution to prevent a bias due to olfactory cues. All behavioral tests (Table 1) were conducted in a manner similar to those previously described [38], [39]. Prior to brain dissection, mice were deeply anesthetized with chloral hydrate (245 mg/kg, i.p.), and the brains were quickly removed. The raw data of behavioral tests, which are not described in this paper, are disclosed in the gene-brain-phenotyping database (http://www.mouse-phenotype.org/).

**Table 1:** Comprehensive behavioral test battery of CD47 KO mice.

Test	Age (w)	Results
Forced Swim Test	12∼18	[21]
General Health	12∼21	Figure 1
Light/Dark Transition Test	12∼21	Figure 5
Open Field Test	12∼21	Figure 5
Social Interaction Test (Crawley’s version)	13∼22	Figure 3
Elevated Plus Maze Test	14∼23	Figure 5
Hot Plate Test	14∼23	Figure 1
Social Interaction Test (novel environment)	14∼23	Figure 4
Rotarod Test	15∼24	Figure 1
Startle Response/Prepulse Inhibition	16∼25	Figure 2
Barnes Maze Test	39∼48	Figure 7
Fear Conditioning Test	43∼52	Figure 7
Gait Analysis Test	44∼53	Figure 1
Tail Suspension Test	44∼53	Figure 6
Social Interaction Test (home cage)	60∼69	Figure 4

Age (w): age in weeks at the beginning of each test.

### Hot Plate Test

The hot plate test was used to evaluate sensitivity to a painful stimulus. Mice were placed on a 55.0 (± 0.3)°C hot plate (Columbus Instruments, Columbus, OH), and latency to the first hind-paw response was recorded with a 15-sec cut-off time. The hind-paw response was defined as either a foot shake or a paw lick.

### Motor Function Tests

A wire hang test apparatus (O’Hara & Co., Tokyo, Japan) was used to assess balance and grip strength. The apparatus consists of a box (21.5×22×23 cm) with a wire mesh grid (10×10 cm) on its top, which can be inverted. The mouse was placed on the wire mesh, which was then inverted, causing the animal to grip the wire. Latency to fall was recorded, with a 60-s cut-off time. A grip strength meter (O’Hara & Co.) was used to assess forelimb grip strength. Mice were lifted and held by their tail so that their forepaws could grasp a wire grid. The mice were then gently pulled backward by the tail with their posture parallel to the surface of the table until they release the grid. The peak force applied by the forelimbs of the mouse was recorded in newtons (N). Each mouse was tested three times and the highest value obtained was used for statistical analysis. Motor coordination and balance were tested with the rotarod test. The rotarod test, using an accelerating rotarod (UGO Basile Accelerating Rotarod, Varese, Italy), was performed by placing mice on rotating drums (3 cm diameter) and measuring the time each animal was able to maintain its balance on the rod. The speed of the rotarod accelerated from 4 to 40 rpm over a 5-min period.

### Gait Analysis

We analyzed gait of adult mice during walk/trot locomotion by ventral plane videography as described [40] using DigiGait Imaging System (Mouse Specifics Inc, Watertown, MA). This system enables mice to walk on a motorized transparent treadmill belt, and the software automatically identifies the stance and swing components of stride, and calculates stance width, stride length, step angle, and paw angle. Briefly, we placed the mice on a treadmill belt that moves at a speed of 24.7 cm/s. We collected digital video images of the underside of mice at 150 frames per second.

### Startle Response/prepulse Inhibition Tests

A startle reflex measurement system (O’Hara & Co.) was used to measure startle response and prepulse inhibition [39]. A test session began by placing a mouse in a Plexiglas cylinder where it was left undisturbed for 10 min. White noise (40 msec) was used as the startle stimulus for all trial types. The startle response was recorded for 140 msec (measuring the response every 1 msec) starting with the onset of the prepulse stimulus. The background noise level in each chamber was 70 dB. The peak startle amplitude recorded during the 140 msec sampling window was used as the dependent variable. A test session consisted of 6 trial types (2 types for startle stimulus only trials and 4 types for prepulse inhibition trials). The intensity of startle stimulus was 110 or 120 dB. The prepulse sound was presented 100 msec before the startle stimulus, and its intensity was 74 or 78 dB. Four combinations of prepulse and startle stimuli (74–110, 78–110, 74–120, and 78–120 dB) were employed. Six blocks of the 6 trial types were presented in pseudorandom order such that each trial type was presented once within a block. The average inter-trial interval was 15 sec (range: 10–20 sec).

### Crawley’s Sociability and Preference for Social Novelty Test

The testing apparatus consisted of a rectangular, three-chambered box and a lid with an infrared video camera (O’Hara & Co.). Each chamber was 20×40×22 cm and the dividing walls were made from clear Plexiglas, with small square openings (5×3 cm) allowing access into each chamber. An unfamiliar C57BL/6 J male (stranger 1), that had had no prior contact with the subject mice, was placed in one of the side chambers. The location of stranger 1 in the left vs. right side chamber was systematically alternated between trials. The stranger mouse was enclosed in a small, round wire cage, which allowed nose contact between the bars, but prevented fighting. The cage was 11 cm in height, with a bottom diameter of 9 cm, vertical bars 0.5 cm apart. The subject mouse was first placed in the middle chamber and allowed to explore the entire test box for a 10-min session. The amount of time spent in each chamber was measured with the aid of a camera fitted on top of the box. Each mouse was tested in a 10-min session to quantify social preference for the first stranger. After the first 10-min session, a second unfamiliar mouse was placed in the chamber that had been empty during the first 10-min session. This second stranger was also enclosed in an identical small wire cage. The test mouse thus had a choice between the first, already-investigated unfamiliar mouse (stranger 1), and the novel unfamiliar mouse (stranger 2). The amount of time spent in each chamber during the second 10-min was measured as described above. Data acquisition and analysis were performed automatically using Image CSI software (see ‘Data analysis’).

### Social Interaction Test in a Novel Environment

In the social interaction test, two mice of identical genotypes that were previously housed in different cages were placed in a box together (40×40×30 cm) and allowed to explore freely for 10 min. Social behavior was monitored with a CCD camera connected to a Macintosh computer. Analysis was performed automatically using Image SI software (see ‘Data analysis’). The total number of contacts, total duration of active contacts, total contact duration, mean duration per contact, and total distance traveled were measured. The active contact was defined as follows. Images were captured at 1 frame per second, and distance traveled between two successive frames was calculated for each mouse. If the two mice contacted each other and the distance traveled by either mouse was longer than 4 cm, the behavior was considered as ‘active contact’.

### Social Interaction Test in Home Cage

Social interaction monitoring in the home cage was conducted as previously described [41]. The system was same apparatus in locomotor activity in home cage. Two mice of the same genotypes that had been housed separately were placed together in a home cage. Their social behavior was then monitored for 9 days. Output from the video camera was fed into a Macintosh computer. Images from each cage were captured at a rate of one frame per second. Social interaction was measured by counting the number of particles detected in each frame: two particles indicated that the mice were not in contact with each other; and one particle (i.e., the tracking software could not distinguish two separate bodies) indicated contact between the two mice. We also measured locomotor activity during these experiments by quantifying the number of pixels that changed between each pair of successive frames. Only eight pairs of mice were used for each genotype due to the limited availability of apparatus. Data acquisition failed in one apparatus, which was measuring the activity of a pair of KO mice. Therefore, we analyzed the data for the remaining eight pairs of WT and seven pairs of KO mice. Analysis was performed automatically using Image HA software (see ‘Data analysis’).

### Open-field Test

Locomotor activity was measured using an open-field test as previously described [39]. Each mouse was placed in the corner of the open-field apparatus (40×40×30 cm; Accuscan Instruments, Columbus, OH). The test chamber was illuminated at 100 lux. Total distance traveled, vertical activity (rearing measured by counting the number of photobeam interruptions), time spent in the center area (20×20 cm), and beam-break counts for stereotypic behaviors were recorded. Data were collected for 120 min.

### Light/dark Transition Test

The light/dark transition test was conducted as previously described [42], [43]. The apparatus used for the light/dark transition test consisted of a cage (21×42×25 cm) divided into two sections of equal size by a partition with a door (O’Hara & Co.). One chamber was made of white plastic and brightly illuminated, whereas the other chamber was black and dark. Mice were placed in the dark side and allowed to move freely between the two chambers with the door open for 10 min. The number of transitions between the two compartments, latency to first enter the lit chamber, distance traveled, and time spent in each chamber were recorded by Image LD4 software (see ‘Data analysis’).

### Elevated Plus-maze Test

The elevated plus-maze test was performed as previously described [44]. The apparatus consisted of two open arms (25×5 cm) and two enclosed arms of the same size, with 15-cm high transparent walls (O’Hara & Co.). The arms and central square were made of white plastic plates and were elevated to a height of 55 cm above the floor. To minimize the likelihood of animals falling from the apparatus, 3-mm high plastic ledges were provided for the open arms. Arms of the same type were arranged at opposite sides to each other. Each mouse was placed in the central square of the maze (5×5 cm), facing one of the closed arms. Mouse behavior was recorded during a 10-min test period. The number of entries into, and the time spent in open and enclosed arms, were recorded. For data analysis, we used the following four measures: the percentage of entries into the open arms, the time spent in the open arms (s), the number of total entries, and total distance traveled (cm). Data acquisition and analysis were performed automatically using Image EP software (see ‘Data analysis’).

### Tail Suspension Test

The tail suspension test was performed for a 10-min test session. Mice were suspended 30 cm above the floor of a white plastic chamber (31×41×41 cm) (O’Hara & Co.) in a visually isolated area by adhesive tape placed ∼1 cm from the tip of the tail, and the behavior was recorded over a 10-min test period. Images were captured at one frame per second. Immobility was judged by the application program according to a certain threshold. Immobility lasting for less than a 2 sec was not included in the analysis. Data acquisition and analysis were performed automatically, using Image TS software (see ‘Data analysis’).

### Contextual and Cued Fear Conditioning Test

Each mouse was placed in a test chamber (26×34×29 cm) inside a sound-attenuated chamber (chamber A; O’Hara & Co.) and allowed to explore freely for 2 min. A 60-dB white noise, which served as the conditioned stimulus (CS), was presented for 30 s, followed by a mild (2 sec, 0.35 mA) footshock, which served as the unconditioned stimulus (US). Two more CS-US pairings were presented with a 2-min inter-stimulus interval. Context testing was conducted 24 hr after conditioning in the same chamber. Cued testing with altered context was conducted after conditioning using a triangular box (35×35×40 cm) made of white opaque Plexiglas, which was located in a different room. The chamber of the test was illuminated at 100 lux. In cued testing with altered context, white noise was delivered from 180 sec and continued until 6 min. Data acquisition, control of stimuli (tones and shocks), and data analysis were performed automatically using Image FZ software (see ‘Data analysis’). Images were captured at 1 frame per second. For each pair of successive frames, the area in which the mouse moved was measured. When this area was below a threshold of 20 pixels, the behavior was judged as ‘freezing’, i.e., complete lack of mobility in any parts of the body during a 1-sec period. When the area equaled to or exceeded the threshold, the behavior was considered ‘non-freezing’. The optimal threshold (amount of pixels) to judge freezing was determined by adjusting it to the amount of freezing measured by human observation. ‘Freezing’ that lasted less than the defined time threshold of 2 sec was not included in the analysis. The parameters were constant for all mice assessed.

### Barnes Maze Test

The Barnes maze test was conducted on ‘dry land’, a white circular surface, 1.0 m in diameter, with 12 holes equally spaced around the perimeter (O’Hara & Co.). The circular open field was elevated 75 cm from the floor. A black Plexiglas escape box (17×13×7 cm) containing paper cage bedding on its floor was located under one of the holes. The hole above the escape box represented the target, analogous to the hidden platform in the Morris task. The location of the target was consistent for a given mouse but was randomized across mice. The maze was rotated daily, with the spatial location of the target unchanged with respect to the visual room cues, in order to prevent bias based on olfactory or proximal cues within the maze. One trial per day for 7 successive days and two trials per day for next 6 successive days were conducted except for no trial on day 6 and one trial on the last day. A probe trial was conducted 24 hr after the last training session without the escape box in order to confirm that this spatial task was performed based on navigation using distal environment room cues. Time of latency to reach the target hole, number of errors, distance to reach the target hole, and time spent around each hole were recorded by Image BM software (see ‘Data analysis’). To assess long-term retention, a second probe trial was applied a week after probe test 1 and additional one session of retraining.

### Data Analysis

The applications used for the behavioral studies (Image LD4, Image SI, Image OF, Image TS, Image SI, Image HA, Image CSI, Image FZ, Image BM) were developed by Dr. Tsuyoshi Miyakawa (available through O’Hara & Co.) based on NIH Image program (NIH, Bethesda, MD, available at http://rsb.info.nih.gov/nih-image/) and ImageJ (ImageJDev.Org, available at http://imagejdev.org/). Statistical analysis was conducted using StatView (SAS Institute, Cary, NC). Data were analyzed using a paired *t*-test, Student’s *t*-test, Mann-Whitney U-test, one-way ANOVA, or two-way repeated measures ANOVA. Values in graphs are expressed as mean ± SEM.

## Supporting Information

Table S1 Behavioral phenotypes in CD47 KO and SIRP-alpha mutant mice.
(XLSX)
